# Mahaim fiber connecting the right atrium to the left ventricle: a case report

**DOI:** 10.1002/joa3.12362

**Published:** 2020-06-01

**Authors:** Daisuke Yakabe, Yusuke Fukuyama, Masahiro Araki, Akemi Aso, Toshihiro Nakamura

**Affiliations:** ^1^ Department of Cardiology Clinical Research Institute National Hospital Organization Kyushu Medical Center Fukuoka City Japan

**Keywords:** accessory pathway, atrioventricular reentrant tachycardia, catheter ablation, left ventricle, Mahaim fiber

## Abstract

In the majority of cases presenting with the Mahaim fiber (MF), the MF connects the lateral right atrium (RA) to the right bundle branch or the right ventricle. We present the case of a 33‐year‐old man with antidromic atrioventricular reentrant tachycardia using MF connected to the septal RA and left ventricle (LV). Although the Mahaim potential was recorded at the septal RA, ablation at this site could not eliminate the MF and had a potential risk of injury to the atrioventricular node. Additional application at the posterior septal LV achieved successful MF ablation.

## Graphical abstract

Mahaim fiber was located between septal right atrium and posterior left ventricle (LV). Successful ablation was achieved at the posterior LV.

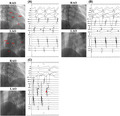

## 1 INTRODUCTION

The Mahaim fiber (MF) is a rare accessory pathway, typically connecting between the lateral wall in the right atrium (RA) and the right bundle branch (RBB) or the right ventricle (RV).^1^ We present a case with an MF connecting the septal RA and the posterior septal left ventricle (LV).

## 2 CASE PRESENTATION

**Figure 1:**
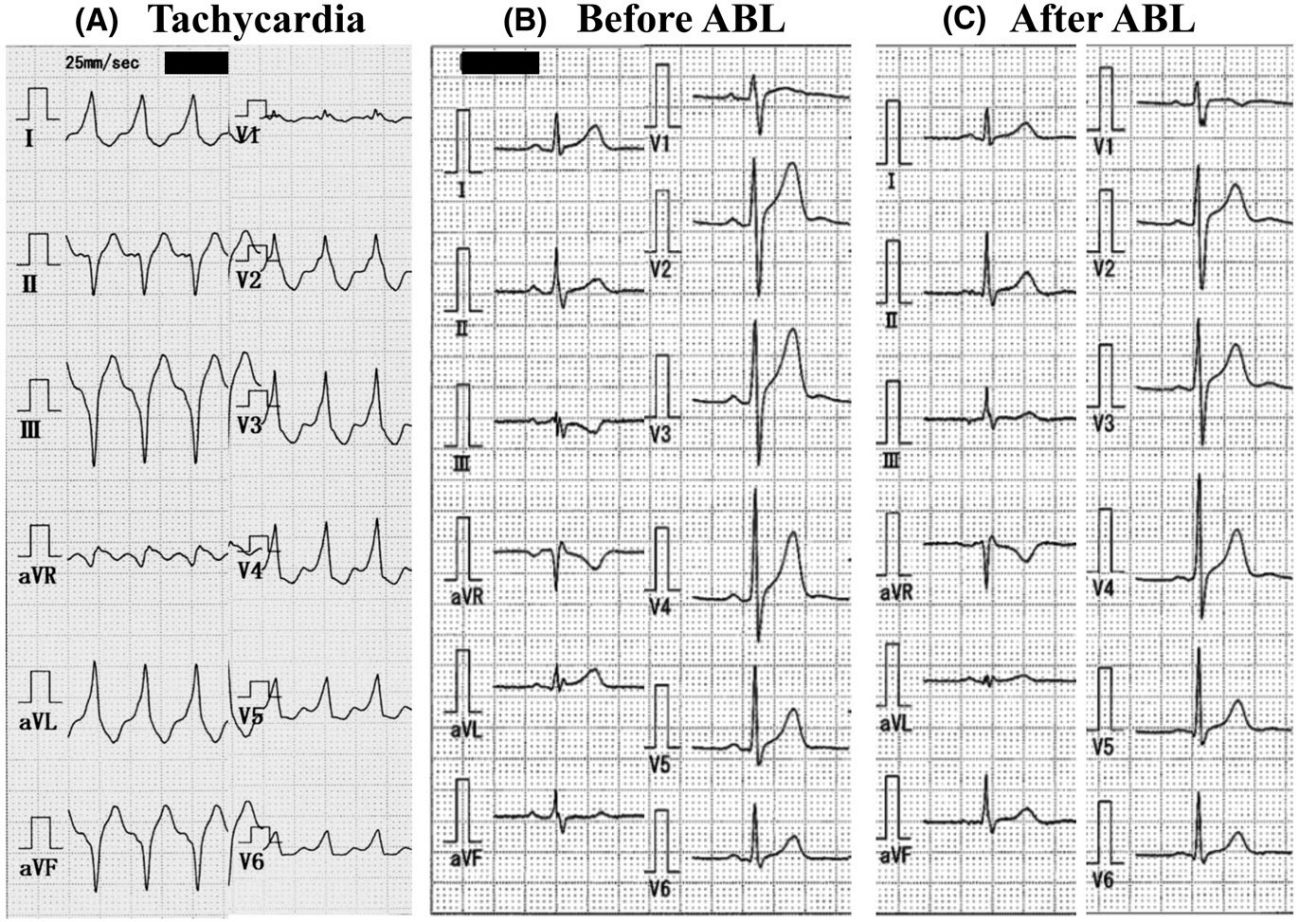
ECG during tachycardia and sinus rhythm. A, ECG during tachycardia shows wide QRS tachycardia with superior axis and positive concordance in precordial leads. B, ECG before catheter ablation shows sinus rhythm without preexcitation. C, ECG after catheter ablation shows the change in R‐wave amplitude at lead III and QRS axis deviated to right side (from 12 to 59°). ABL, ablation

A 33‐year‐old man had been experiencing sudden‐onset palpitations for 14 years. Two months before admission, the patient experienced palpitations and visited a nearby clinic. A 12‐lead ECG showed wide QRS tachycardia of right and left bundle branch block morphology and a superior axis, which was restored to sinus rhythm with intravenous verapamil. No delta wave or shortened PQ interval was observed during sinus rhythm (Figure 1). Results from physical examination and other testing revealed no abnormalities. The patient was referred to our hospital for a catheter ablation.

An electrophysiological study was conducted after informed written consent was obtained. Multielectrode catheters were placed in the high RA (HRA), His bundle (HB), tricuspid valve annulus (TVA), RBB, coronary sinus (CS), and RV. During sinus rhythm, AH and HV intervals were 76 and 38 milliseconds, respectively. Incremental atrial pacing and atrial extrastimulation revealed prolongation of the AH interval, shortening of the HV interval, and progressive preexcitation (Figure S1). The morphology of the QRS complex was identical to that in clinical tachycardia during maximal preexcitation. Clinical tachycardia was induced by continuous atrial pacing. The earliest atrial activation site was at the HB during tachycardia, which was the same during ventricular pacing. The earliest ventricular activation site was the ostial CS, not HB or RBB. Ventricular pacing and extrastimulation during sinus rhythm revealed concentric retrograde decremental conduction. Para‐Hisian pacing revealed the absence of other retrograde accessory pathways. Tachycardia terminated with VA block by a single stimulus from the RV during HB refractoriness. While tachycardia was reset with a single stimulus from the ostial CS, tachycardia was not reset from the HRA and lateral TVA. These findings implied that the tachycardia circuit was located at the septal RA, not at the lateral RA. Atrioventricular nodal reentrant tachycardia (AVNRT) was also induced during this study. The HA interval during clinical tachycardia was equal to that during ventricular pacing, and was longer than the HA interval during AVNRT, suggesting that AVNRT with bystander MF could be ruled out (Figure S2). Finally, this tachycardia was diagnosed as antidromic atrioventricular reentrant tachycardia (AVRT) using anterograde MF and retrograde AVN conduction.

**Figure 2:**
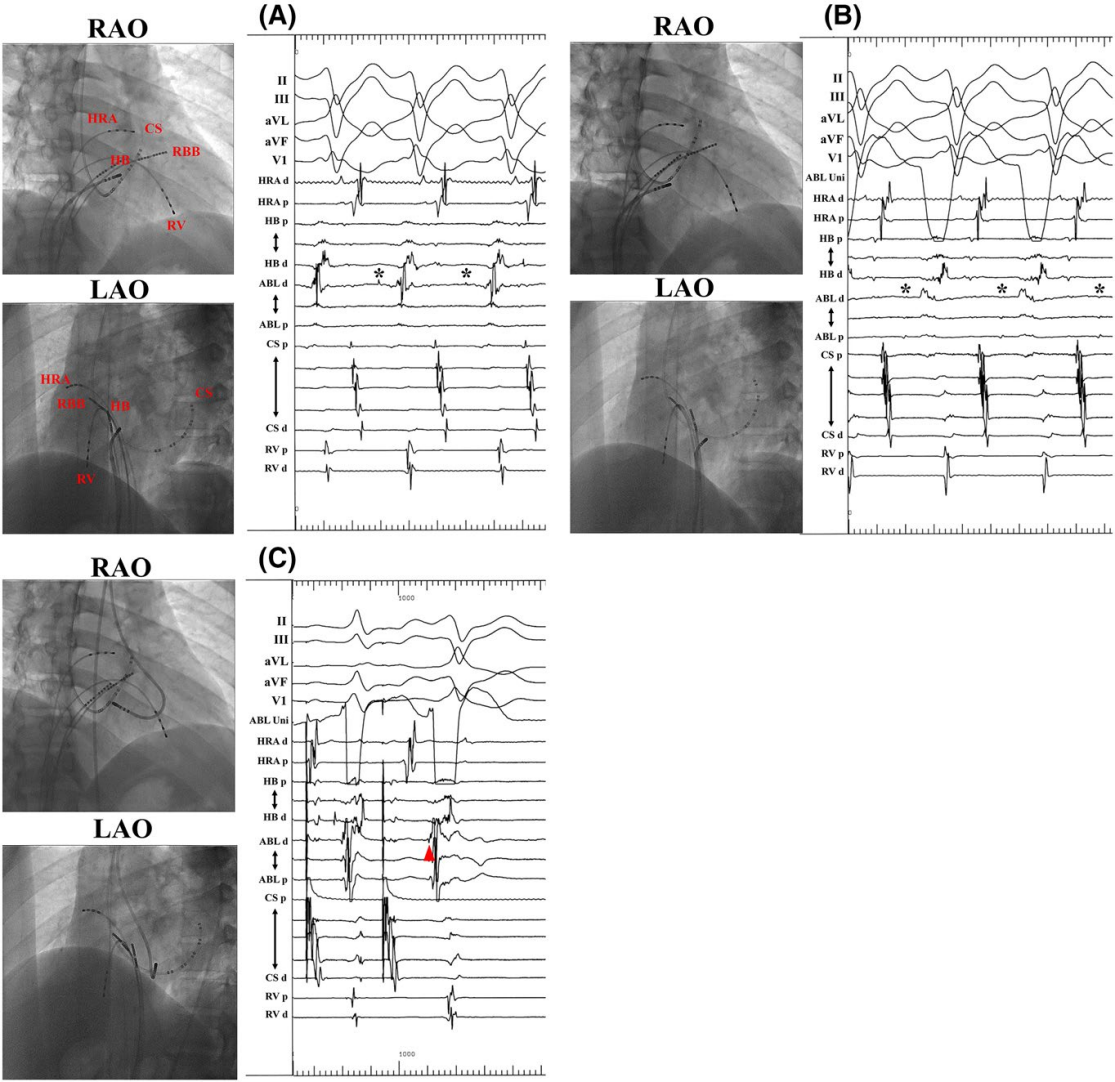
Catheter positions and intracardiac electrograms. Mahaim potential (asterisk) was clearly recorded at mid‐septal RA during tachycardia (A), however, its amplitude was lower at the posterior septum (B). Successful elimination was achieved at this site with earliest ventricular electrogram during maximal preexcitation (red arrow), regardless of the absence of Mahaim potential (C). ABL, ablation; CS, coronary sinus; d, distal; HB, His bundle; HRA, high right atrium; LAO, left anterior oblique; LV, left ventricle; p, proximal, RA, right atrium; RAO, right anterior oblique; RBB, right bundle branch; RV, right ventricle

The Mahaim potential (MP) was mapped using a 4‐mm tip nonirrigated ablation catheter. The MP was clearly recorded at the mid‐septal RA during programmed atrial pacing and tachycardia (Figure 2A). There was no MP at the lateral RA, RBB, RV, or ostial CS. Because radiofrequency application at this site might pose a risk of atrioventricular block (AVB), the target site was shifted to the posterior side (Figure 2B). A QS pattern on unipolar electrogram of the ablation catheter was observed at this site; nevertheless, the elimination of MF failed. Therefore, the ablation catheter was introduced to the basal LV through a transaortic approach. The earliest ventricular activation site during programmed atrial pacing was identified at the posterior septal LV (Figure 2C). Single radiofrequency application terminated tachycardia. After ablation, atrial pacing did not show any findings of MF and tachycardia could not be induced. Twelve‐lead ECG after ablation showed that the amplitude of the R wave at lead III increased and the QRS axis deviated to the right side (Figure 1C). He had no symptoms for 10 years after catheter ablation.

## 3 DISCUSSION

In 1941, Mahaim et al first reported a peculiar accessory pathway having three characteristics: (a) anterograde decremental conduction properties, (b) no retrograde conduction, and (c) a prolonged AH interval and a shortened HV interval, with manifestation of the delta wave during atrial pacing. Further research revealed that the MF is a muscular fiber with a distinct atrial‐ventricular connection, which extends from the lateral TVA side at the atrial edge to the RBB or RV at the ventricular edge.^1^

The "left Mahaim" is extremely rare. Although some investigators have reported that the left MF is located at the free wall of the mitral annulus (MA)^2^ and the posterior septal MA,^3^, ^4^, ^5^ the etiology of “left Mahaim” is unknown. Tada et al^4^ reported a case where the MF was eliminated at the posterior‐septal MA. Hluchy et al.^5^ reported a two‐case series; one case of successful ablation at the septum RA and the other case of the septal MA. In our case, MF may be located at the septal RA and connected with the posterior septal LV because (a) MP was clearly recorded at the septal RA, rather than at the lateral RA, (b) the earliest ventricular site during tachycardia was at the posterior septal LV, and (c) successful MF ablation could be achieved at this site. This report is novel with regard to presentation of MF with an RA‐LV connection. A potential limitation in this case is that we did not use a three‐dimensional mapping system. Further investigation should be needed to identify the accurate location.

## 4 CONCLUSION

We report a case of antidromic AVRT via MF connecting RA and LV. When radiofrequency application at the septal RA has potential risks of AVB, careful mapping and ablation at LV should be considered.

## CONFLICT OF INTEREST

Authors declare no conflict of interests for this article.

## Supporting information

Appendix Fig S1

Appendix Fig S2

Supplementary Material
