# Supplementary figures and images for: Mahaim fiber connecting the right atrium to the left ventricle: a case report
Source: J Arrhythm. 2020 Jun 1;36(4):774–6. doi: 10.1002/joa3.12362 (PMC7411216; doi:10.1002/joa3.12362)

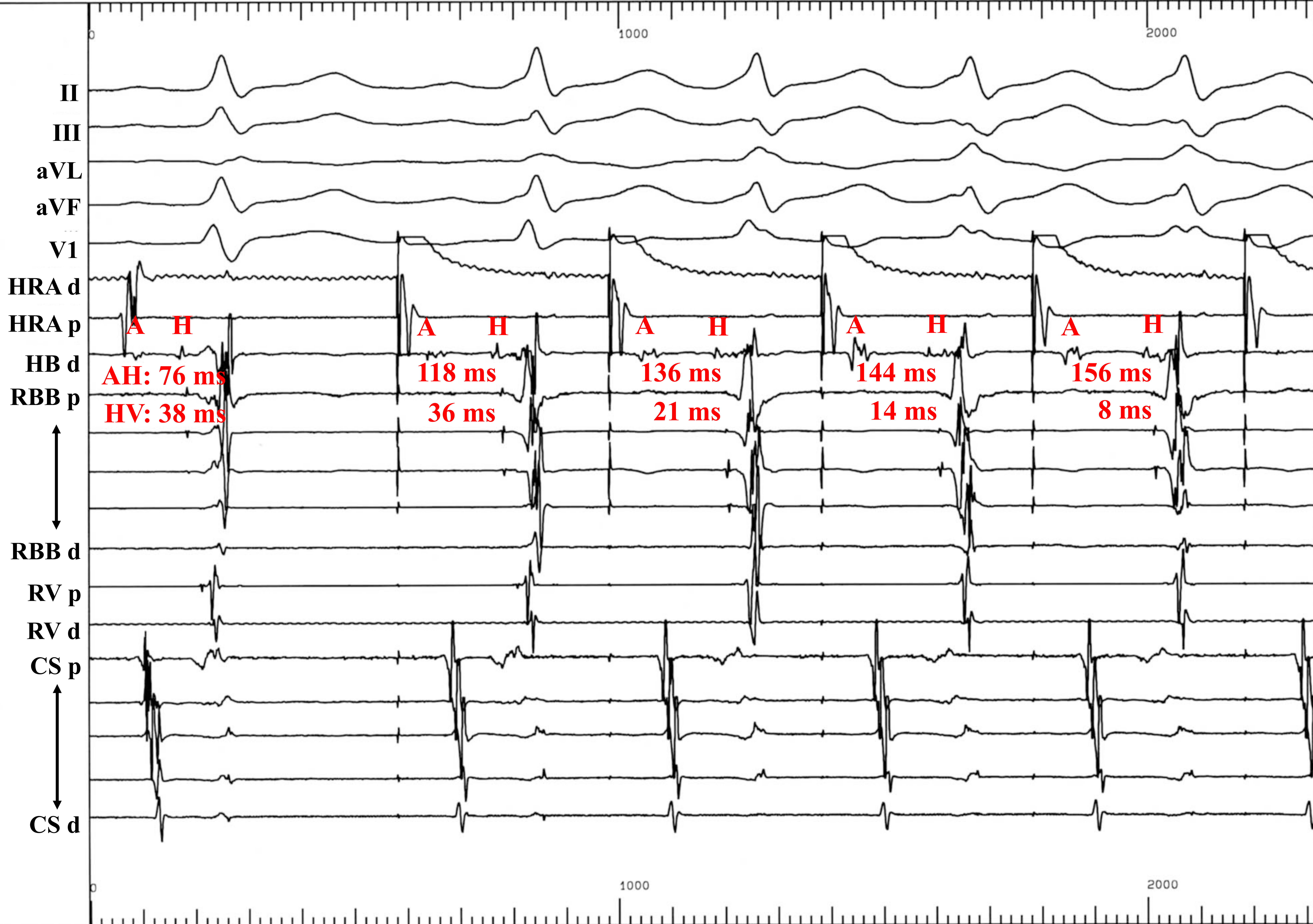

Supplement: Supplementary file 1 — Appendix Fig S1 [file JOA3-36-774-s001.pdf]

**(a) AVRT**

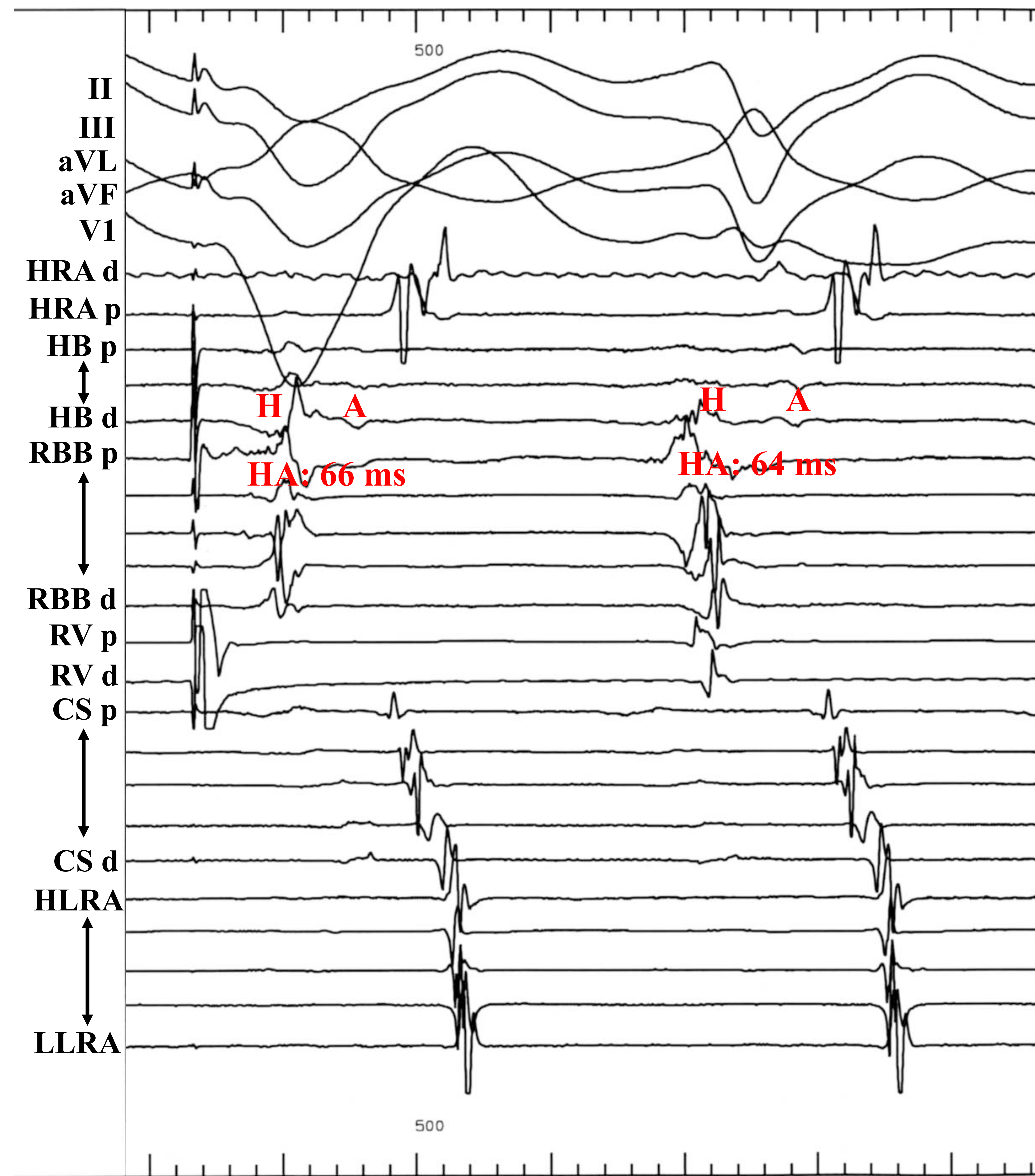

**(b) AVNRT**

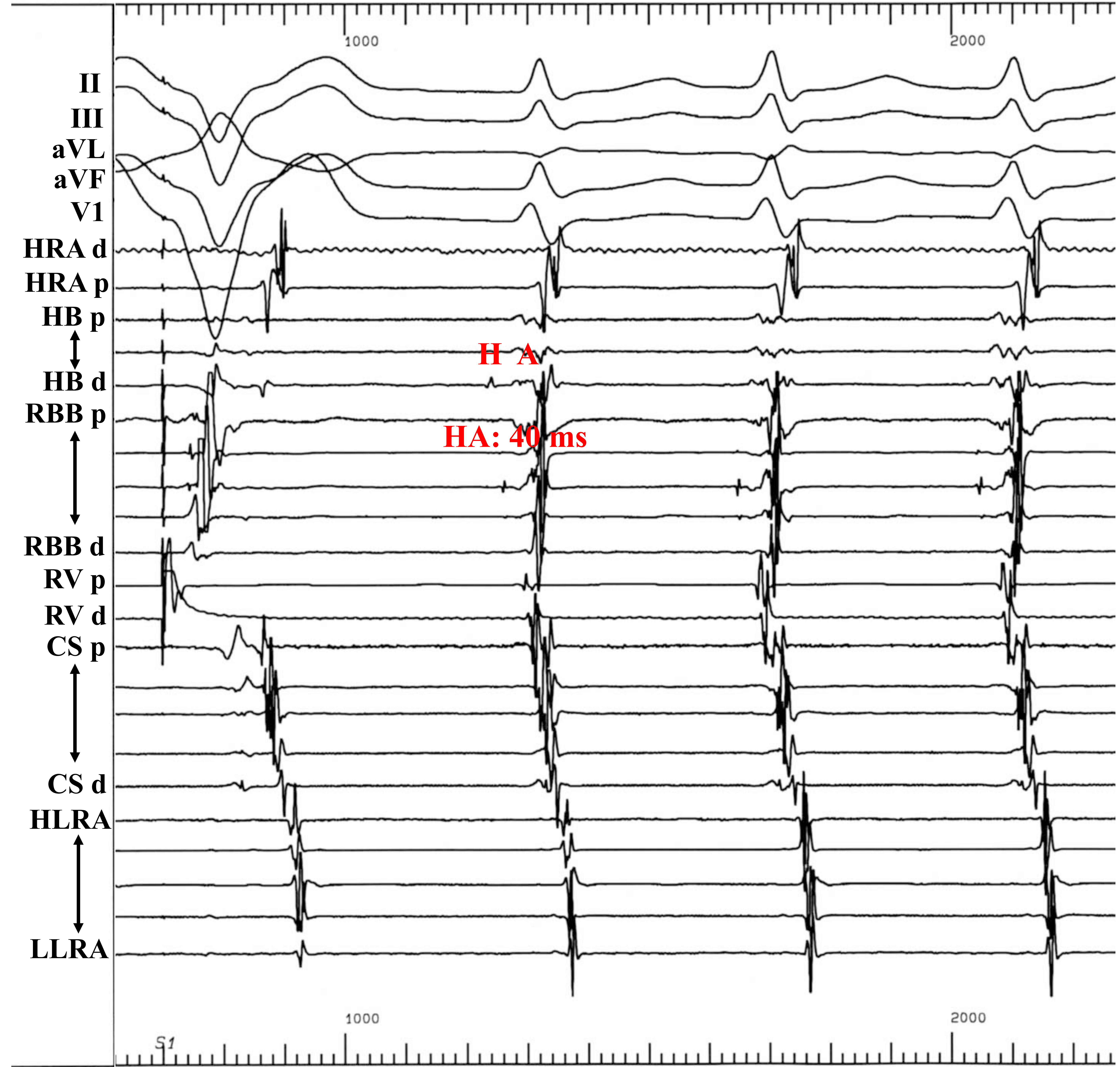

Supplement: Supplementary file 2 — Appendix Fig S2 [file JOA3-36-774-s002.pdf]
