# Supplementary material for: Mahaim fiber connecting the right atrium to the left ventricle: a case report
Source: J Arrhythm. 2020 Jun 1;36(4):774–6. doi: 10.1002/joa3.12362 (PMC7411216; doi:10.1002/joa3.12362)
Supplement: Supplementary file 3 — Supplementary Material [file JOA3-36-774-s003.docx]

**Appendix (figures)**

**Appendix Figure 1.** Progressive preexcitation during atrial pacing

Prolongation of the AH interval, shortening of the HV interval, and manifested preexcitation were shown during atrial pacing at 150 per minute. Earliest ventricular activation site was ostial CS during pacing.

CS, coronary sinus; d, distal; HB, His bundle; HRA, high right atrium; p, proximal; RBB, right bundle branch; RV, right ventricle.

**Appendix Figure 2.** Intracardiac electrograms during AVRT, ventricular pacing, and AVNRT.

The HA intervals during ventricular pacing and AVRT (a) were longer than that during AVNRT (b).

AVNRT, atrioventricular nodal reentrant tachycardia; AVRT, atrioventricular reentrant tachycardia; CS, coronary sinus; d, distal; HB, His bundle; HLRA, high lateral right atrium; HRA, high right atrium; LLRA, low lateral right atrium; p, proximal; RBB, right bundle branch; RV, right ventricle.
